# The Relationship between Working Night Shifts and Depression among Nurses: A Systematic Review and Meta-Analysis

**DOI:** 10.3390/healthcare11070937

**Published:** 2023-03-24

**Authors:** Chidiebere Emmanuel Okechukwu, Corrado Colaprico, Sofia Di Mario, Agbonvihele Gregrey Oko-oboh, David Shaholli, Maria Vittoria Manai, Giuseppe La Torre

**Affiliations:** 1 Department of Public health and Infectious Diseases, Sapienza University of Rome, Piazzale Aldo Moro 5, 00185 Rome, Italy; 2 Health Sciences Unit, Tampere University, 33520 Tampere, Finland

**Keywords:** circadian rhythm, sleep, nurses, shift work, mental health, depression

## Abstract

Background: For many years, occupational physicians have debated whether there is a link between working the night shift and depression and other co-occurring mental health issues, with an emphasis on work-related, biological, individual, and environmental factors. We performed this systematic review and meta-analysis to estimate the overall association between sleep deprivation and depression among nurses working night shifts. Methods: A systematic search was carried out across the electronic databases PubMed, Scopus, and Web of Science from inception to 30 September 2022, for studies that reported a relationship between estimated night shift work and depression in nurses. The outcomes were measured using the odds ratio (OR) and matching 95% confidence interval (CI). The I^2^ statistic was used to assess heterogeneity. The Grading of Recommendations Assessment, Development and Evaluation technique was used to evaluate the quality of the evidence, and the Newcastle–Ottawa Scale was utilized to assess the methodological quality of each of the included studies. We determined the overall relationship between working nights and the onset of depression. Results: A total of 20 studies were included in the systematic review. Furthermore, 8 studies were included in the meta-analysis due to their common use of the OR as an effect measure. The 8 studies gave an overall estimate indicating a statistically significant association between night shift work and depression among nurses (OR = 1.49 95% CI: 1.26, 1.76). The prediction interval for the overall estimate was (0.995, 2.231). This implies that the true OR in a future study would most likely fall within this range, with a 95% certainty. Conclusions: The outcome of this systematic review and meta-analysis showed a significant association between night shift work, the circadian and sleep disruption it causes, and the risk of depression in nurses. This demonstrates that nurses who work night shifts are at risk of developing depression.

## 1. Introduction

There is a lot of data showing that shift work, especially night employment, is unhealthy. Since humans are diurnal creatures, their typical sleep–wake cycle entails sleeping at night and being awake during the day [1]. The night shift is frequently included in the shift schedules that nurses follow. Nurses must be awake and vigilant in order to offer patients the best care possible. Nurses who work night shifts may get exhausted and sleep deprived, which could endanger the safety of patients [2]. The condition of not getting enough time or a good enough night’s sleep to sustain reasonable alertness, performance, and health is known as sleep deprivation, often referred to as sleep insufficiency or sleeplessness [3]. Night shift work significantly increases the prevalence of sleep deprivation due to disruption of the sleep–wake cycle, which is associated with an altered sequence of activity and rest. Insomnia means difficulty falling or staying asleep that is accompanied by daytime impairments related to those sleep troubles [3]. Sleepiness or drowsiness, is a state of strong desire for sleep, or sleeping for unusually long periods. It can refer to the condition of being in a drowsy state due to circadian rhythm disorders or it can be a symptom of other health problems [4]. It can be accompanied by lethargy, weakness, and a lack of mental agility. The excessive sleepiness and impaired driving performance resulting from shift work increase the risk of motor vehicle crashes by two to four times during the commute to and from work [5]. Shift work can result in circadian disruptions that are associated with cardiometabolic diseases, cancers, gastrointestinal health issues, and the worsening of mood disorders and depressive symptoms [6]. Shift work is associated with depressive symptoms and may exacerbate symptoms in individuals with a history of depression and mood disorders [7]. According to the World Health Organization, depression is a common mental disorder [8]. It is characterized by persistent sadness and a lack of interest or pleasure in previously rewarding or enjoyable activities. It can also disturb sleep and appetite. Tiredness and poor concentration are common. Depression is a leading cause of disability around the world and contributes greatly to the global burden of disease. The effects of depression can be long-lasting or recurrent and can dramatically affect a person’s ability to function and live a rewarding life [8]. The impact of shift work on sleep and health has been examined in the past. Based on the findings of a 2017 integrated review, the researchers were unable to say for sure if nurses who work shifts performed psychologically worse than those who do not. Instead, they argue that some nurses may have negative psychological effects from working shifts, and that these effects may be strongly influenced by both environmental and personal factors [9].

Disrupted circadian rhythm and insufficient sleep quality and quantity have been recognized as two of the most significant factors in the long-term impacts of night shift work on nurses’ mental health [10]. Both factors can reduce nurses’ alertness and cognitive performance during the night shift [11]. One study found that women who work shifts, especially night shifts, are more likely than men to have depressive symptoms [12]. Since women make up the majority of the nursing workforce, this is a serious concern. However, the coronavirus pandemic served as a wake-up call for hospital administrators and nurse managers to recognize nurses’ susceptibility to mental health issues like depression and sleep issues, as well as how the two are related, in order to develop effective strategies to reduce the psychological burden of nurses working various shifts [13]. The objective of this study was to systematically summarize and quantitatively evaluate some selected cross-sectional, case–control, and cohort studies in order to determine the relationship between night shift work and the risk of depression in nurses. Interestingly, no systematic review and/or meta-analysis has been conducted in the past to specifically determine the relationship between night shift work and the risk of depression in nurses.

## 2. Methods

A protocol, explaining the objectives, search strategies, inclusion and exclusion criteria, outcomes and methods of analysis was developed before the implementation of this study, and it was successfully registered on PROSPERO (CRD42022354487). The protocol adheres to the Preferred Reporting Items for Systematic Review and Meta-Analysis Protocols guidelines [14]. The inclusion criteria were that any selected study must be an experimental study, a case–control study, a cross-sectional study, or a longitudinal study that quantitatively estimated the relationship between night shift work and depression in nurses. Any study that did not comply with the aforementioned criteria was excluded. The criteria for conducting a meta-analysis were whether at least three results were available and whether the outcome measures could be pooled.

## 3. Literature Search Strategy

We systematically searched three reputable academic databases for experimental studies, case–control studies, cross-sectional studies, and cohort studies that quantitatively estimated the relationship between night shift work and depression in nurses. PubMed, Scopus, and Web of Science were searched from onset to September 2022. We combined the keywords, which are Medical Subject Headings terms, to search the databases as described in Table 1. Study selection and the removal of duplicates were managed using Zotero. For the selection of studies, two reviewers were involved (C.E.O, and S.M).

## 4. Study Selection and Data Extraction

We included studies that reported an association between sleep deprivation and/or circadian rhythm disruption and depression among nurses working night shifts. Studies were excluded if the researchers investigated an unrelated topic, used an uncategorized population, or had insufficient data. Reviews, conference abstracts, editorial letters, qualitative research, animal model papers, and duplicate reports were excluded. If there were duplicate studies, the most recent or complete version was selected. If multiple languages were used to describe and publish the same data, the English version was selected. We extracted data on the first author, year of publication, country of origin, study design, participant numbers and characteristics, exposure, and outcomes and their measurement tools. The data from all the included studies was extracted and independently reviewed by C.E.O, D.S, C.C, and S.D.M. Any dispute or disagreement between the two reviewers that could not be agreed upon was resolved by M.V.M, and G.L.

## 5. Assessment of Risk of Bias and Grading the Evidence

The methodological quality of all the included studies was independently assessed using a modified version of the Newcastle–Ottawa Scale (NOS) [15]. Potential risk of bias was identified and assessed using the 13-item version of the Research Triangle Institute Item Bank tool, which is a development of the original 29-item questionnaire [16]. The 13-item Research Triangle Institute Item Bank is a tool for observational studies assessing selection, confounding factors, performance, attrition, detection, and reporting biases, with high reliability. The items were scored as having a high, low, unclear, or not applicable risk of bias. Each study was assigned an overall risk of bias score based on the proportion of high and valid items and a cut-off of 50%. The degree of confidence in the estimate for the outcome was determined by the Grading of Recommendations Assessment, Development, and Evaluation approach (GRADE) [3]. The rating criteria were based on the risk of bias, inconsistency, indirectness, imprecision, publication bias, and other considerations. The overall quality of the evidence in the included studies was moderate.

## 6. Outcomes and Statistical Analysis

Our main interest was to estimate the relationship between sleep deprivation and depression among nurses working night shifts. The association between sleep and depression was expressed as the odds ratio (OR). We obtained the ORs and corresponding 95% confidence intervals (CIs) from most of the included studies. The statistical analysis was conducted using RevMan 5.4.1 (Cochrane Collaboration, 2020). Outcomes were assessed by a pooled logOR among all included studies, and the corresponding 95% CIs were calculated by a fixed effects model or a random effects model. The prediction interval (PI) was calculated using the PI program in the Comprehensive Meta-Analysis Software. Statistical heterogeneity was defined by I^2^ value ≥ 50%. Potential publication bias was assessed by testing the asymmetry of the funnel plot.

## 7. Results

### 7.1. Study Identification and Selection

A total of 2074 studies were identified through a preliminary database search, and 411 duplicates were excluded by Zotero. By browsing the titles and abstracts, 1663 studies were available and entered full-text screening. After reading these documents carefully, 1630 studies were excluded (1020 contained ambiguous data that could not be extracted, 328 were reviews or conference abstracts, 49 were editorial letters, 182 were qualitative research studies, and 51 full-text articles could not be obtained).

**Figure 1:**
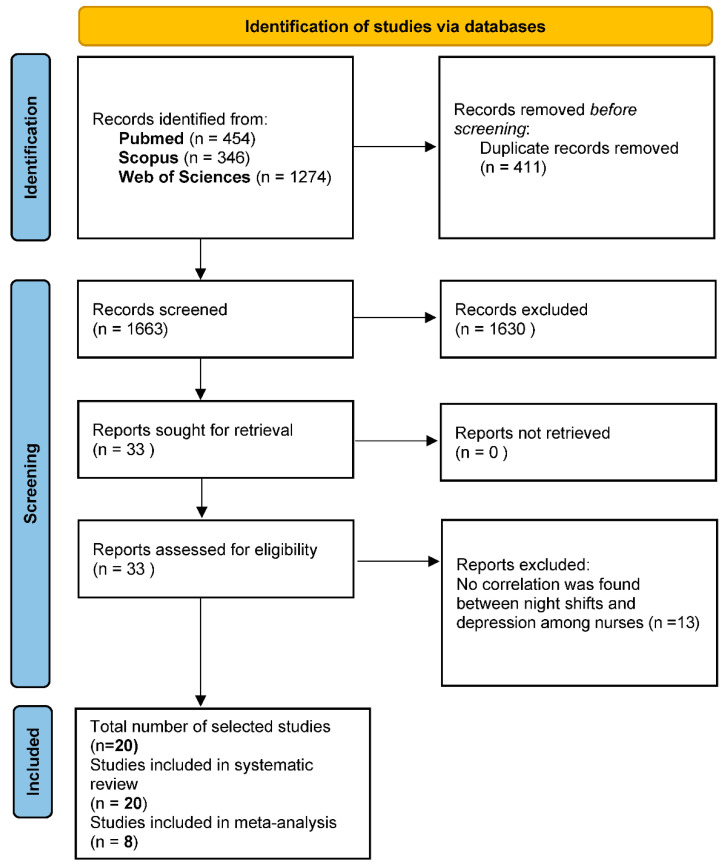
PRISMA flow diagram of selected studies.

Finally, a total of 20 eligible studies were included in the systematic review and 8 studies in this meta-analysis. Figure 1 shows the detailed research identification and selection process.

### 7.2. Study Characteristics

The 20 studies included were published between 1990 and 2022, covering a total of 37,983 participants. There were 18 cross-sectional studies and 2 cohort studies. The sample sizes in each study varied from 39 to 11,450, and most participants were registered nurses working various shifts. The majority of the studies (n = 9) used the Hospital Anxiety and Depression Scale to assess depression among the nurses. This is a self-assessment scale that has been developed and found to be a reliable instrument for detecting states of depression and anxiety. The characteristics of the included studies are shown in Table 2. Appendix A shows the differences in the characteristics of the 20 studies with respect to population, sex and age distribution, exposure and comparator variables, outcome measures, and study design. Except for one study, which sampled nurses, firefighters, and government workers, all the other studies recruited only nurses. Five studies recruited only female nurses. The mean age of the study population ranged from 25.3 to 50 years. However, three studies did not state the age of their study population. All studies assessed depression as the outcome variable using different tools. Although seven studies had a similar definition of night shift as the exposure of interest, the other studies had varied definitions of shift work, with some incorporating the intensity and frequency of the shifts over time. The comparison group was also defined differently; nine studies used day shift workers, while one used the general population. Appendix B summarizes the statistical output of the studies, including the effect measures, variations in effects, strength of association, and heterogeneity. Eight studies measured the association between night shift work and depression using the odds ratio; five used the mean difference, and four used the regression coefficient. Eleven studies performed further statistical analysis to adjust for confounding variables; this included five of the eight studies that used the odds ratio as an effect measure. Additionally, eleven studies reported a statistically significant association between night shift work and depression.

### 7.3. Outcome of Risk of Bias and Quality Assessment

**Figure 2:**
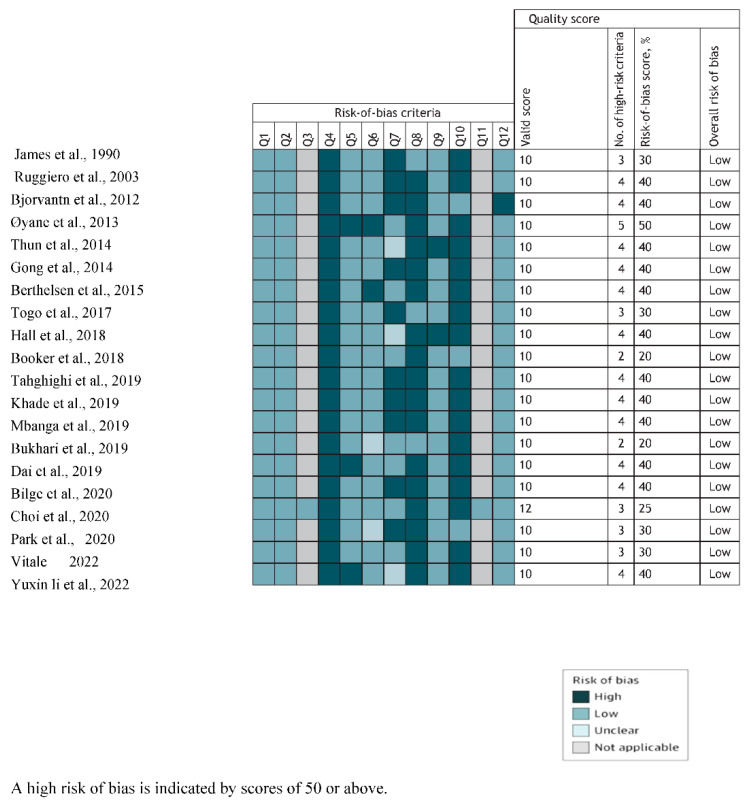
The included studies’ risk of bias as determined by the Research Triangle Institute Item Bank [17,18,19,20,21,22,23,24,25,26,27,28,29,30,31,32,33,34,35,36].

The risk of bias in the included studies was evaluated using the 13-item version of the Research Triangle Institute Item Bank tool. The risk-of-bias assessment was performed by two authors (C.O. and G.O.) independently, with discrepancies being resolved by discussion and, if needed, by a third author (D.S.). The items were scored as having a high, low, unclear, or not applicable risk of bias. Each study received an overall risk of bias score based on the ratio of high and valid items (excluding not applicable items) and an arbitrary cutoff of 50%. The quality of the included articles was assessed using the NOS. Green highlighted articles have 9 stars or more, indicating high quality; yellow highlighted articles have 7 or 8 stars, indicating moderate quality; and red highlighted articles have 6 stars or fewer, indicating low quality (see Appendix C). Six of the cross-sectional studies in our review are of high quality, eleven are of moderate quality, and one is of low quality. From the cohort studies, one study has high quality and the other has low quality. The outcomes for risk of bias assessment and quality assessment in the overall study and for each study are shown in Figure 2 and Appendix C, respectively.

## 8. Meta-Analysis and Outcome

**Figure 3:**
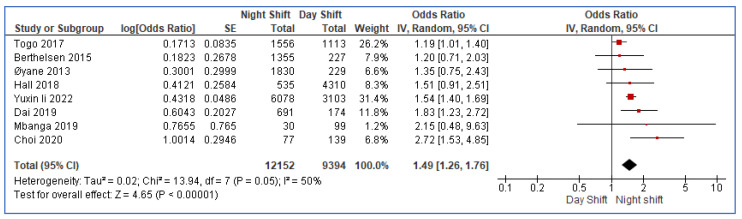
Forest plot of the summary of odds ratio of comparable studies [20,23,24,25,29,31,33,36].

Figure 3 shows the forest plot of the individual and summary estimates of the eight studies included in the meta-analysis. All the tools used in the eight included studies in the assessment of depression were tools that were already validated, and the researchers also endeavored to validate the tools. The percentage of females in the population and mean age in the included studies for the meta-analysis are summarized in Appendix D. These eight studies were included in the meta-analysis due to their common use of the odds ratio (OR) as an effect measure. The random effects model was used to test for heterogeneity among the eight studies. Overall, the model was statistically significant (*p* < 0.00001). A 50% heterogeneity was estimated using the I^2^ at *p* = 0.05. This indicates low heterogeneity among the eight studies. The individual studies’ effects ranged from an odds ratio of 1.19 (1.01, 1.40) to 2.72 (1.53, 4.85). All individual effect estimates were to the right of the line of no effect (OR = 1). However, the confidence intervals of five studies cut across the line of no effect. The 7th study had the widest confidence interval and was thus the least precise, while the 5th study had the smallest confidence interval, making it the most precise. The 5th study also contributed the most weight (31.4%) to the summary estimate, while the 7th contributed the least (1.2%). The inverse variance method was used for weighting the studies. We discovered that nurses who work night shifts had a significant risk of depression after combining the ORs reported in the eight studies, yielding an overall estimate of 1.49 (1.26, 1.76). The PI for the overall estimate was (0.995, 2.231). We would expect that in about 95% of all populations, the true OR will fall in this range.

## 9. Publication Bias

**Figure 4:**
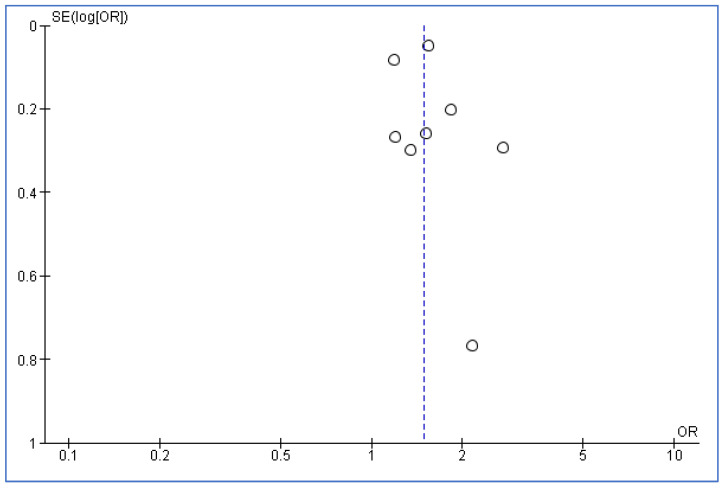
Funnel plot to explore publication bias.

Given the studies that were included in the meta-analysis, Figure 4 is a funnel plot to investigate the publication bias. No signs of publication bias were seen in the way the included studies were symmetrically dispersed on each side of the overall effect line.

## 10. GRADE Assessment of All Included Studies

The overall quality of the evidence of all included studies in this review, as shown in Table 3, was graded as moderate for the association between sleep disturbance and depression among nurses working night shifts. Moreover, there was less heterogeneity among selected studies, and no publication bias was discovered.

## 11. Discussion

**Figure 5:**
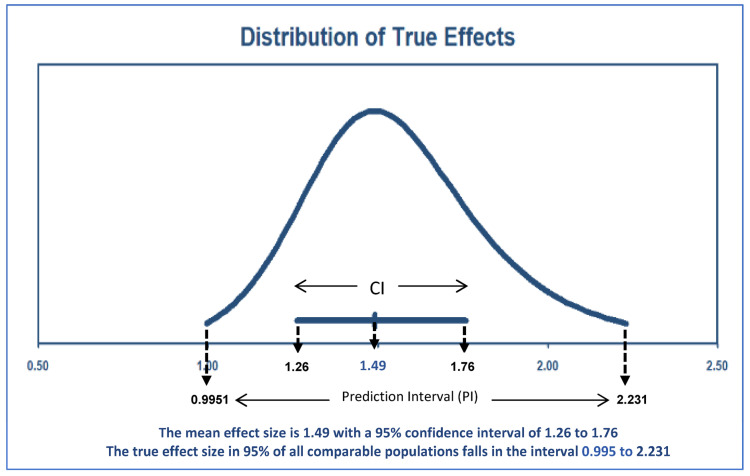
95% confidence interval (CI) and Prediction Interval (PI) for the effect estimate.

This study was conducted to estimate the association between sleep deprivation and depression among nurses working night shifts. At the end of the literature search, 20 articles were selected, and there was a total population of 37,983 participants, and 8 studies were included in the meta-analysis. At the end of the statistical analysis, the main results demonstrated that there was a significant increased risk of developing depression for nurses working night shifts. Shift work increased the total risk of negative mental health outcomes (such as depression and anxiety) by 28% among 28,431 workers, according to a study by Torquati et al. [12]. Humans by nature follow a sleep–wake cycle that involves sleeping during the night and waking during the day [10]. However, to maintain the smooth operation of the healthcare sector, many nurses and other healthcare professionals and non-healthcare staff employed in a healthcare setting work during the night either rotationally or permanently [10]. Nurses, for example, work in the night to enable continuous care and monitoring of patients throughout the night and to administer medication to them. Regularly working the night shift, however, can cause sleep deprivation, which has been associated with cognitive problems, mood alterations, reduced job performance, reduced motivation, increased safety risks, and physiological changes [37]. According to the findings reported in the studies by Thun et al. [21] and Berthelsen et al. [23], nurses who changed from night work to day work reported a significant decrease in symptoms of anxiety and depression from baseline to 2-year follow-up. From the outcome of our review, it also emerged that depression is often accompanied by sleep disorders, particularly insomnia, as found in the studies of Øyane et al. [20] and Khade et al. [28]. This result is partially consistent with that of one study, which found that nurses were at higher risk of developing some subtypes of insomnia [38]. In the study of Bilge et al. [32], it was pointed out that nurses who worked night shifts had higher rates of depression and sexual dysfunction. A study by Huang et al. [39] found that nurses do not appear to seek medical attention for some types of disorders, particularly anxiety and depression, even though nurses may be more susceptible to stress-related psychiatric problems. The nurses’ personal attitudes toward such disorders may be a contributing factor to the reasons why nurses are reluctant to seek care for psychological or behavioral health problems [39]. The barriers appear to be that getting help for mental health concerns can be more explicitly stigmatizing in terms of what their friends and employers might think of them, which could have negative effects on their career advancement [40,41]. Depression and anxiety were discovered in 58.82% and 62.08% of shift nurses, respectively, according to the findings of the study by Li et al. [36]. These rates were influenced by fatigue during shift work, psychological stress before/during/after night shifts, feeling energetic after resting before/after night shifts, using sleep medication before/after night shifts, physical discomfort during night shifts, being occupied during night shifts, and food intake during shift work. They suggested that minimizing shift nurses’ workloads, preventing the causes of stress during night shifts, and promoting rest and relaxation could help with depression and anxiety [36]. Nursing managers should increase rest days after night shifts, increase night shift spacing, and decrease overtime in order to lower the prevalence of shift work sleep disorders among nurses [42]. The findings from our study agree with those of previous meta-analyses conducted in different populations. Considering sociodemographic factors, the outcome of subgroup meta-analysis by gender, night shift work duration, type of occupation, and continent carried out by Lee et al. [43] showed that night shift work was consistently associated with an increased risk of depression. Moreover, a meta-analysis of five studies revealed a 42% increase in the risk of depression among persons working the night shift, as conducted by Angerer et al. [44], but the researchers believe that this evidence is not strong enough to sustain a general medical recommendation against night shift work for workers with depressive conditions and that it would seem appropriate to address this question on an individual basis, with strong support from physicians and close attention to the negative psychosocial factors associated with night shift work. According to one study in China, night shift work, shift frequency, and sleep disturbances were associated with an increased risk of depression among workers, and the association between night shift work and depression appeared to be partially mediated by sleep disturbances [45]. According to the Heinz Nixdorf Recall Study, which investigated various demographic, lifestyle, and occupation-correlated features to observe the relationship between shift work and depressive symptoms, women who work nights had higher relative risks of developing depressive symptoms, particularly when working night shifts for 20 years or more [46]. Working night shifts consistently increased the likelihood of women developing depression symptoms, according to stratified analysis [46]. In this study, we included the PI in order to help in the interpretation of the heterogeneity by estimating the expectations from new observations in future studies and giving the range within which, the results of a future study might lie. The PI program in the Comprehensive Meta-Analysis Software included the CI in the PI graph. The width of the horizontal line around the estimate is the range of the CI. The beginning and the end of the curve indicate the PI. The footnote attached to the graph further indicated the numerical values and ranges of both the CI (1.26–1.76) and the PI (0.995–2.231) (see Figure 5). The PI of 0.995–2.231 obtained from the meta-analysis suggests that, if a new study were to be conducted, the true OR in that study is likely to fall within this range with 95% certainty. There will be some populations where the effect size will be suggestive of no difference (the lower limit contains the null value of 1.00). This interval considers both the within-study sampling error and the between-study heterogeneity in the meta-analysis, as well as any additional variability that may arise due to differences in the population or study design in the new study.

## 12. Strengths and Limitations

From the outcome of this study, we can see that circadian rhythm disruption and sleep deprivation should be considered as clinical risk factors for depression in nurses working night shifts. Circadian and sleep disruptions should be among the therapeutic targets in order to prevent and treat depression among nurses working night shifts and in the planning of occupational health interventions for nurses. One of the major limitations of our review was that it included studies that assessed night shift work effects based on working rotating shifts and casual night shifts and, therefore, could not demonstrate a strong reciprocity of associations between working night shifts and depression. Furthermore, all the included studies were either cross-sectional or cohort studies, so the underlying pathophysiological mechanisms were difficult to ascertain. Additionally, there was a lack of uniform diagnostic criteria and diagnostic tools as the authors used diverse assessment tools for depression, which might reduce the comparability between studies. Lastly, the pooled effect sizes of the studies selected for the meta-analysis were small.

## 13. Conclusions

The outcome of this study clearly shows a significant relationship between working night shifts, the disturbance of sleep and circadian rhythm linked to night work, and the risk of depression in nurses. This indicates that nurses who work night shifts are more likely to experience depression. A thorough examination of whether sleep deprivation caused by working night shifts significantly increases the incidence of depression in nurses may aid in the development of strategies to improve nurses’ performance during night or rotating shifts, as well as effective shift rota planning. Examples of such strategies include improving workplace standards, personalizing work schedules according to individuals’ circadian rhythm, which will synchronize their biological clock with a schedule, and treating sleep problems and depression among nurses.

## Figures and Tables

**Table 1:** Literature search strings used in the databases.

Database	Search String	Total Number of Results
Web of science	((nurses) AND night shift) AND depression ((nurses) AND rotating shift) AND depression ((nurses) AND shiftwork) AND depression (((nurses) AND wellbeing) AND night shift work AND mental health) (((nurses) AND occupational stress) OR burnout AND shift work AND psychological health) (((nurses) AND circadian rhythm) AND mental health)	1274
Scopus	((nurses) AND night shift) AND depression ((nurses) AND rotating shift) AND depression ((nurses) AND shiftwork) AND depression (((nurses) AND wellbeing) AND night shift work AND mental health) (((nurses) AND occupational stress) OR burnout AND shift work AND psychological health)	346
PubMed	((nurses) AND night shift) AND depression ((nurses) AND rotating shift) AND depression ((nurses) AND shiftwork) AND depression (((nurses) AND wellbeing) AND night shift work AND mental health) (((nurses) AND occupational stress) OR burnout AND shift work AND psychological health) (((nurses) AND circadian rhythm) AND mental health)	454

**Table 2:** Characteristics of included studies.

N.	First Author	Year	Country	Study Design	Sample Size	Assessment Tool for Depression	Aim of the Study	Main Outcomes
1	James [17]	1990	United States	Cross-sectional study	463	Hospital Anxiety and Depression Scale	To determine the relationships between physical health and depression among nurses working shifts.	There was no significant correlation between shift work and signs of depression among nurses.
2	Ruggiero [18]	2003	United States	Cross-sectional study	142	Beck Depression Inventory-II	To evaluate shift-related variations in chronic fatigue and the influences of sleep quality, anxiety, and depression among female nurses.	Depression and poorer sleep quality were more prevalent in nurses that work night shifts than those that work day shifts.
3	Bjorvantn [19]	2012	Norway	Cross-sectional study	267	Hospital Anxiety and Depression Scale	To investigate sleep, sleepiness, fatigue, subjective health complaints (musculoskeletal, gastrointestinal), anxiety, and depression in a sample of Intensive Care Unit nurses.	21.5% and 14.8% of the Intensive Care nurses scored above the cut-off values for anxiety and depression, respectively.
4	Øyane [20]	2013	Norway	Cross-sectional study	2059	Hospital Anxiety and Depression Scale	To determine the relationship between working the night shift with sleep problems and psychological wellbeing.	There was a significant positive correlation between insomnia and depression among night shift nurses.
5	Thun [21]	2014	Norway	Cross-sectional study	633	Hospital Anxiety and Depression Scale	To compare the developmental trajectories of anxiety and depressive symptoms among nurses working night shifts (night only or a three-shift schedule) with those of nurses working shifts that do not include nights (day only or a two-shift schedule).	Compared to day workers, nurses who switched from night to day shifts had a significant decline in anxiety and depressive symptoms over time.
6	Gong [22]	2014	China	Cross-sectional study	3474	Chinese version of the 20-item Zung Self-Rating Depression Scale	To investigate the association between working conditions and depressive symptoms among nurses using a cross-sectional study.	1320 nurses reported having depressive symptoms, making the prevalence of depressive symptoms around 38% overall. A total of 20.82% of the nurses worked two or more night shifts each week.
7	Berthelsen [23]	2015	Norway	Cohort study	2059	Hospital Anxiety and Depression Scale	To investigate factors that may lead to shift work disorder in Norwegian nurses.	A reduction in the number of night shifts or cessation of working night shifts was associated with a decrease in depression and shift work disorder.
8	Togo [24]	2017	Japan	Cross-sectional study	2669	The Center for Epidemiologic Studies Depression Scale	To determine the relationships between depressive symptoms, morningness–eveningness, sleep duration, and rotating shift work among nurses.	Depression was more prevalent in rotating shift nurses compared to nurses who work only day shifts.
9	Hall [25]	2018	Canada	Cross-sectional study	11,450	Composite International Diagnostic Interview Short Form, Major Depression section	To investigate the relationships between work rota and depression among Canadian nurses.	There was a strong relationship between work rota and depression, which was very apparent among rotating and regular night shift nurses.
10	Booker [26]	2018	Australia	Cross-sectional study	202	Patient Health Questionnaire	To assess the relationship between shift work disorder risk, depression, and anxiety using validated tools.	Nurses who were at serious risk of shift work disorder had higher depression.
11	Tahghighi [27]	2019	Australia	Cross-sectional Study	1369	Depression, Anxiety, and Stress Scale—21 Items	To compare nurses who work shifts with nurses who work regular hours in order to comprehend the effects of shift work on psychological health and resilience.	There was relatively high levels of depression among both shift and non-shift nurses, but there was no significant difference between the two groups.
12	Khade [28]	2019	India	Cross-sectional study	190	Hospital Anxiety and Depression Scale	To determine the relationship between working the night shift with sleep problems and psychological wellbeing.	There was a significant positive correlation between insomnia and depression among night shift nurses.
13	Mbanga [29]	2019	Cameroon	Cross-sectional study	143	9-item Patient Health Questionnaire	To determine the prevalence and predictors of depression among nurses in Cameroon.	Nurses who are working night shifts reported more depression and poorer sleep quality than day nurses.
14	Bukhari [30]	2019	Pakistan	Cross-sectional study	250	Agha Khan University Anxiety and Depression Scale	To determine the factors that were associated with depression among Pakistani nurses.	The prevalence of depression in nurses working rotating shifts was significantly higher.
15	Dai [31]	2019	China	Cross-sectional study	865	Hospital Anxiety and Depression Scale	To compare sleep quality as well as depressive symptoms in nurses working night shifts to those working day shifts only.	Among the 865 nurses, 353 (40.8%) were considered to have depressive symptoms. The logistic regression analysis demonstrated that night work was independently associated with the presence of depressive symptoms.
16	Bilge [32]	2020	Turkey	Cross-sectional study	163	Beck Depression Inventory	To determine the effect of nurses’ way of working on depression indicators and their sexual lives.	Nurses who worked night shifts had higher rates of depression and higher rates of sexual dysfunction.
17	Choi [33]	2020	South Korea	Cross-sectional study	77	Hospital Anxiety and Depression Scale	To examine the progression of depressed and anxiety symptoms in nurses working night shifts (night only or a three-shift schedule) with nurses working shifts without nights.	Depressive mood and anxiety symptoms were significantly worse in nurses working three-shift schedule.
18	Park [34]	2020	South Korea	Cross-sectional study	39	Hospital Anxiety and Depression Scale	To propose the mediating role of grey matter structures in the relationship between sleep disturbance and depressive symptoms.	All day-working nurses reported depressive symptoms within the normal range, whereas 39% of the shift-working nurses reported mild depression beyond the normal range.
19	Vitale [35]	2022	Italy	Cohort study	408	Depression, Anxiety, and Stress Scale—21 Items	To evaluate variations in body mass index features, shift, work history, and nurses’ levels of stress, anxiety, and depression.	There was no significant difference in the rate of depression between night shift nurses and their day shift colleagues.
20	Yuxin li [36]	2022	China	Cross-sectional study	11,061	9-item Patient Health Questionnaire	To evaluate and describe the mental health status of Chinese nurses, including symptoms of depression and anxiety, while focusing on the effects of shift work-related characteristics.	Shift work correlated with higher levels of depression among all nurses.

**Table 3:** GRADE assessment of evidence quality.

Criteria	No of Studies	Study Design	Risk of Bias	Inconsistency	Indirectness	Imprecision	Publication Bias	Other Considerations	No of Subjects	Relative (95% CI)	Overall Quality of Evidence	Importance
Nurses, shift work, and depression	20	Cross-sectional studies and cohort studies	Low	Significant	Not significant	Not significant	Not significant	None	37,983	-	⊕⊕⊕⊝ Moderate	Critical

## Data Availability

Not applicable.

## Appendix A. Clinical Heterogeneity of Included Studies

**No**	**Author**	**Year**	**Population**	**Sex**	**Exposures**	**Comparator**	**Outcomes**	**Study Design**	**Mean Age (Years)**
1	James [17]	1990	Nurses	B	Night shift	Other shifts	depression	cross-sectional	35
2	Ruggiero [18]	2003	Nurses	F	Night shift	Day shift	depression	cross-sectional	44.9
3	Bjorvantn [19]	2012	Nurses	B	Shift work	General population	depression	cross-sectional	39.4 (9.1)
4	Øyane [20]	2013	Nurses	B	Current night shift	No night shift	depression	cross-sectional	33.1 (8.2)
5	Thun [21]	2014	Nurses	F	Night shift	Day shift	depression	cross-sectional study	33.1 (8.2)
6	Gong [22]	2014	Nurses	B	Night shift (>=2/week)	Night shift (<2/week)	depression	cross-sectional	31.93 (7.55)
7	Berthelsen [23]	2015	Nurses	B	Night shift (current/previous)	No night shift	depression	cohort study	30–39
8	Togo [24]	2017	Nurses	B	Rotating shift	Day shift	depression	cross-sectional	Day worker = 41.5 (9.7), Rotating shift = 40.2 (10.4)
9	Hall [25]	2018	Nurses	B	High-precision work schedule (regular days)	High-precision work schedule (Rapid frequency rotating shifts)	depression	cross-sectional	45–54
10	Booker [26]	2018	Nurses	B	High-risk SWD	Low-risk SWD	depression	cross-sectional	35.28 (12.02)
11	Tahghighi [27]	2019	Nurses	B	Shift work	Non-shift work	depression	cross-sectional	Shift work = 46.8 (11.50), Non-shift work = 50.0 (9.99)
12	Khade [28]	2019	Nurses	B	Number of night shifts in last 1 month	None	depression	cross-sectional	25.3 (5.4)
13	Mbanga [29]	2019	Nurses	B	Number of night shifts in last 1 week	None	depression	cross-sectional	29.8 (6.55)
14	Bukhari [30]	2019	Nurses	F	Rotating shift	Not stated	depression	cross-sectional	Not stated
15	Dai [31]	2019	Nurses	B	Night shift	Day shift	depression	cross-sectional	Not stated
16	Bilge [32]	2020	Nurses	F	Night shift	Day shift	depression	cross-sectional	36.0 (6.37)
17	Choi [33]	2020	Nurses, Firefighters, Day workers	B	Nurses working 3-shift schedule	Other day workers	depression	cross-sectional	Nurses—28.7 (5.13), Firefighters—42.8 (9.38), Day workers—40.1 (8.30)
18	Park [34]	2020	Nurses	F	Shift work	Day shift	depression	cross-sectional	Shift work—28.1 (3.2), Day shift—30.8 (4.6)
19	Vitale [35]	2022	Nurses	B	Night shift	Day shift	depression	cross-sectional	Not stated
20	Yuxin li [36]	2022	Nurses	B	Shift work	No shift work	depression	cross-sectional	33.24 (7.31)
B: both male and female, F: female only.									

## Appendix B. Methodological Heterogeneity of Included Studies

**No**	**Author**	**Year**	**Effect Measure**	**Study Group**	**Comparator Group**	**OR (95% CI)/B/r/π/*p*-Value**	**Statistical Adjustment**	**Direction of Effect**
1	James [17]	1990	Regression coefficient	NA	NA	B = 0.09	not done	negative
2	Ruggiero [18]	2003	Mean difference	10.35 (7.89)	7.18 (6.65)	*p* < 0.01	not done	positive
3	Bjorvantn [19]	2012	Mean difference	5.0 (2.3)	3.5 (3.1)	*p* < 0.001	not done	positive
4	Øyane [20]	2013	Odds ratio	Not stated	Not stated	1.35 (0.75–2.42)	done	negative
5	Thun [21]	2014	Regression coefficient	NA	NA	B = −0.05	done	negative
6	Gong [22]	2014	Regression Coefficient	NA	NA	B = 1.53	done	positive
7	Berthelsen [23]	2015	Odds ratio	Not stated	Not stated	1.20 (0.71–2.03)	not done	negative
8	Togo [24]	2017	Cohen’s d/odds ratio	550/1006	351/762	Cohens’ d = 0.084	not done	positive
9	Hall [25]	2018	Odds ratio	Not stated	Not stated	1.51 (0.91–2.51)	done	negative
10	Booker [26]	2018	Mean difference/R-squared	7.54 (4.28)	3.78 (3.24)	R2 = 0.184	done	positive
11	Tahghighi [27]	2019	Mean difference	Not stated	Not stated	*p* = 0.514	done	negative
12	Khade [28]	2019	Correlation coefficient	Not stated	Not stated	not stated	not done	negative
13	Mbanga [29]	2019	Odds ratio	Not stated	Not stated	1.58 (1.01–2.48)	done	positive
14	Bukhari [30]	2019	Proportional difference	Not stated	Not stated	*p* = 0.012	not done	positive
15	Dai [31]	2019	Odds ratio	Not stated	Not stated	1.83 (1.23–2.72)	done	positive
16	Bilge [32]	2020	Mean difference	Not stated	Not stated	*p* < 0.001	done	positive
17	Choi [33]	2020	Mean difference/Odds ratio	7.64 (3.35), 41/36	6.30 (3.64), 41/98	*p* = 0.014	not done	positive
18	Park [34]	2020	Mean difference	43.1 (6.4)	35.3 (4.9)	*p* = 0.0003	not done	positive
19	Vitale [35]	2022	Regression coefficient	NA	NA	B = −0.019	done	negative
20	Yuxin li [36]	2022	Odds ratio	2305/3773	1515/1588	1.54 (1.4–1.69)	done	positive
OR (95% CI): odds ratio (95% confidence interval), B: regression coefficient, r: correlation coefficient, π: prevalence/proportion, NA: not applicable.								

## Appendix C. Newcastle–Ottawa Quality Assessment Scale for the Cross-Sectional and Cohort Studies Included in the Systematic Review and Meta-Analysis

**Author (Year)**	**Selection**	**Comparability**	**Exposure/Outcome**
**Cross-Sectional studies (*n* = 18)**			
James, 1990 [17]	*****	*	***
Bjorvantn, 2012 [19]	****	**	***
Øyane, 2013 [20]	*****	**	***
Hall, 2018 [25]	*****	*	***
Booker, 2018 [26]	*****	*	***
Park, 2020 [34]	*****	*	***
Ruggiero, 2003 [18]	*****	*	**
Gong, 2014 [22]	*****	*	**
Thun, 2014 [21]	*****	*	**
Togo, 2017 [24]	*****	*	**
Dai, 2019 [31]	*****	*	**
Khade, 2019 [28]	*****	*	**
Mbanga, 2019 [29]	*****	*	**
Tahghighi, 2019 [27]	*****	*	**
Bilge, 2020 [32]	*****	*	**
Choi, 2020 [33]	*****	*	**
Yuxin li, 2022 [36]	*****	*	**
Bukhari, 2019 [30]	**	*	**
**Cohort studies (*n* = 2)**			
Berthelsen, 2015 [23]	*****	**	***
Vitale, 2022 [35]	*****	*	**
* = star. Green highlight = 9 stars or more. Yellow highlight = 7 or 8 stars. Red highlight = 6 stars or fewer.			

## Appendix D. Percentage of Female Population and Mean Age in the Included Studies for the Meta-Analysis

**No**	**Author**	**Year**	**Population**	**Sex**	**Outcomes**	**Study Design**	**Tools**	**Validation**	**% Female**	**Mean Age (y)**
1	Øyane [20]	2013	Nurses	B	depression	cross-sectional	HADS	Yes	90.6	33.1
2	Berthelsen [23]	2015	Nurses	B	depression	cohort study	HADS	Yes	91	34.5
3	Togo [24]	2017	Nurses	B	depression	cross-sectional	CES-D	Yes	96.1	40.7
4	Hall [25]	2018	Nurses	B	depression	cross-sectional	CIDI-SFMD	Yes	94.7	41.5
5	Mbanga [29]	2019	Nurses	B	depression	cross-sectional	PHQ-9	Yes	67.1	29.8
6	Dai [31]	2019	Nurses	B	depression	cross-sectional	HADS	Yes	98	29.2
7	Choi [33]	2020	Nurses, Day workers	B	depression	cross-sectional	HADS	Yes	55.1	36.0
8	Yuxin li [36]	2022	Nurses	B	depression	cross-sectional	PHQ-9	Yes	94.8	33.2
B = Both males and females, HADS = Hospital Anxiety and Depression Scale, CES-D = Center for Epidemiologic Studies Depression Scale, CIDI-SFMD = Composite International Diagnostic Interview Short Form, Major Depression section, PHQ-9 = 9-item Patient Health Questionnaire.										
